# Serum BAFF and GDF11: cross-sectional associations with glycolipid metabolism and subclinical carotid artery wall thickening in elderly type 2 diabetes patients

**DOI:** 10.3389/fcvm.2026.1891416

**Published:** 2026-08-18

**Authors:** Xiaojuan Wang, Ye Yao, Menghua Liu, Tingting Pei, Weini Chen, Xiaoli Nie

**Affiliations:** 1 Department of Nephrology, Southern Medical University Hospital of Integrated Traditional Chinese and Western Medicine, Southern Medical University, Guangzhou, China; 2 School of Traditional Chinese Medicine, Hunan University of Medicine, Huaihua, China; 3 Key Laboratory of Drug Metabolism Research and Evaluation of the State Drug Administration, Guangdong Provincial Key Laboratory of New Drug Screening, School of Pharmaceutical Sciences, Southern Medical University, Guangzhou, China; 4 Department of Nephrology, The Affiliated Traditional Chinese Medicine Hospital, Guangzhou Medical University, Guangzhou, China; 5 Department of Endocrinology, Zhuhai Hospital of Integrated Traditional Chinese and Western Medicine, Zhuhai, China; 6 Department of Nephrology and Rheumatology, Guangdong Provincial Hospital of Integrated Traditional Chinese and Western Medicine, Foshan, China

**Keywords:** aged, B-Cell activating factor, carotid intima-Media thickness, diabetes mellitus, type 2, growth differentiation factor 11, insulin resistance

## Abstract

**Background:**

Subclinical carotid artery wall thickening (SCWT) is a macrovascular complication in elderly patients with type 2 diabetes mellitus (T2DM), linked to chronic inflammation and metabolic dysregulation. The cytokines B-cell activating factor (BAFF) and growth differentiation factor 11 (GDF11) are involved in immune-metabolic pathways, but their combined associations with glycolipid metabolism and SCWT in elderly T2DM patients are not well characterized. This study aimed to investigate these associations.

**Methods:**

In this cross-sectional observational study, 205 elderly patients with T2DM and 150 age-matched healthy controls were enrolled. Serum concentrations of BAFF, GDF11, and glycolipid parameters were measured. Carotid intima-media thickness (cIMT) was assessed via ultrasound in the T2DM group only, with SCWT defined as cIMT > 1.2 mm. Statistical analyses included Pearson correlation and multivariate logistic regression.

**Results:**

Compared to controls, T2DM patients had higher levels of BAFF, FPG, 2hPPG, HbA1c, TC, TG, and LDL-C, and lower levels of GDF11 and HDL-C (all *P* < 0.001). BAFF levels correlated positively with FPG, 2hPPG, HbA1c, TC, TG, and LDL-C, and negatively with HDL-C, while GDF11 showed the opposite correlation pattern (all *P* < 0.05). SCWT was present in 87 patients (42.4%). The SCWT subgroup had higher BAFF and lower GDF11 than T2DM patients without SCWT (both *P* < 0.001). Multivariate analysis showed that longer diabetes duration, higher HOMA-IR, and elevated BAFF were independently associated with increased odds of SCWT, while higher GDF11 was associated with decreased odds.

**Conclusion:**

Elevated BAFF and reduced GDF11 are associated with dysregulated glycolipid metabolism and with the presence of subclinical carotid artery wall thickening in this elderly T2DM cohort. These findings suggest potential roles for these cytokines in diabetic macrovascular pathology.

## 1 Introduction

Type 2 diabetes mellitus (T2DM) represents a global epidemic of dysregulated metabolism, characterized by chronic hyperglycemia and insulin resistance. Its prevalence escalates markedly with age, making the elderly a particularly vulnerable demographic due to compounded effects of declining physiological function and comorbid conditions (1). Beyond microvascular sequelae, T2DM accelerates macrovascular disease, with subclinical carotid artery wall thickening (SCWT) being a common complication (2, 3). SCWT is associated with ischemic cerebrovascular events and contributes to morbidity and mortality in the aging diabetic population (2, 3). Despite advances in glycemic control, the burden of macrovascular complications remains high, underscoring the need for improved risk stratification and novel therapeutic targets.

The pathogenesis of SCWT in T2DM is multifactorial, involving both metabolic derangement and chronic low-grade inflammation (4). Persistent hyperglycemia and insulin resistance lead to dyslipidemia, characterized by elevated triglycerides (TG), low-density lipoprotein cholesterol (LDL-C), and reduced high-density lipoprotein cholesterol (HDL-C), which provides a substrate for arterial lipid accumulation (5). Concurrently, an immune-inflammatory response is activated, which may damage the vascular endothelium, promote foam cell formation, and contribute to plaque development (6, 7). Despite this understanding, the molecular mediators linking the diabetic milieu to carotid artery wall thickening remain incompletely elucidated. In particular, the roles of immune-derived cytokines in bridging metabolic dysregulation and vascular remodeling are not well characterized.

B-cell-activating factor (BAFF), a member of the tumor necrosis factor (TNF) superfamily, regulates B lymphocyte survival, proliferation, and immunoglobulin production. Beyond its role in adaptive immunity, BAFF has been associated with metabolic and vascular inflammation (8). Elevated BAFF levels have been reported in autoimmune conditions and, more recently, in cardiometabolic disease. In T2DM, BAFF has been implicated in insulin resistance and dyslipidemia (8). It may amplify inflammatory cascades that impair endothelial function and contribute to arterial wall thickening (9). Clinically, higher serum BAFF levels have been correlated with cardiovascular events in non-diabetic cohorts (10) and with carotid intima-media thickness in systemic lupus erythematosus (11). However, the role of BAFF in SCWT in elderly patients with T2DM remains undefined.

Growth differentiation factor 11 (GDF11), a circulating member of the transforming growth factor-*β* superfamily, has been studied for its potential protective effects in cardiovascular diseases (12–14). Preclinical studies suggest that GDF11 may improve glucose homeostasis by enhancing pancreatic *β*-cell function and reducing apoptosis (12, 13), ameliorate myocardial ischemia-reperfusion injury (15), and promote endothelial progenitor cell mobilization and neovascularization (16). In animal models, GDF11 has been associated with reduced inflammatory cell recruitment and smaller atherosclerotic lesions (17). It has been postulated to mitigate oxidative stress and improve endothelial progenitor cell function (18). However, data on circulating GDF11 levels in human cardiometabolic diseases are varied, with some studies reporting lower levels in diabetes (8). Whether GDF11 is associated with SCWT in elderly T2DM patients is unknown.

BAFF and GDF11 have not been investigated together in relation to glycometabolic control and SCWT. We hypothesized that in elderly patients with T2DM, there may be an imbalance characterized by higher BAFF and lower GDF11 that may correlate with glycolipid metabolism and be associated with SCWT. To explore this hypothesis, we conducted a cross-sectional observational study with the following objectives: The primary objective was to evaluate the associations of serum BAFF and GDF11 with SCWT, alongside traditional risk factors, within the T2DM cohort. The secondary objectives were (1) to compare serum levels of BAFF and GDF11 between elderly T2DM patients and healthy controls; and (2) to analyze their correlations with indices of glucose and lipid metabolism. The control group was included to establish whether BAFF and GDF11 levels are altered in the setting of glycolipid dysregulation, thereby highlighting the potential relevance of these biomarkers in diabetic metabolic disturbances.

## 2 Materials and methods

### 2.1 Study design and participants

**Figure 1:**
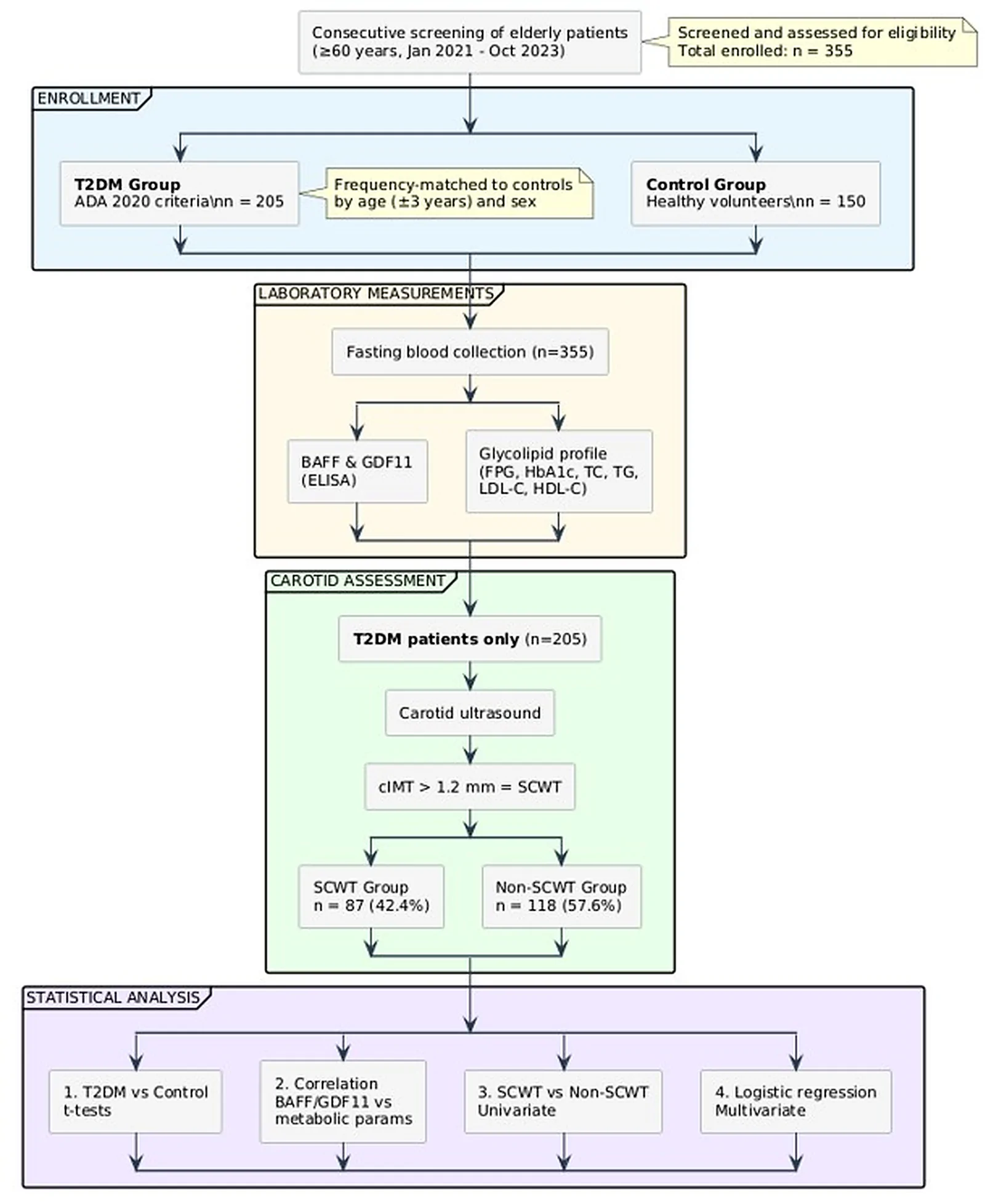
Study flowchart: participant enrollment, measurements, and analysis. T2DM, type 2 diabetes mellitus; SCWT, subclinical carotid artery wall thickening; cIMT, carotid intima-media thickness; BAFF, B-cell activating factor; GDF11, growth differentiation factor 11; FPG, fasting plasma glucose; TC, total cholesterol; TG, triglycerides; LDL-C, low-density lipoprotein cholesterol; HDL-C, high-density lipoprotein cholesterol.

A cross-sectional observational study was conducted at Zhuhai Integrated Traditional Chinese and Western Medicine Hospital between January 2021 and October 2023. The study protocol was reviewed and approved by the Ethics Committee of Southern Medical University Hospital of Integrated Traditional Chinese and Western Medicine, Guangzhou, China (Approval No. E20210123). All procedures were conducted in accordance with the ethical standards of the institutional committee and the 1964 Declaration of Helsinki and its later amendments. All participants provided written informed consent. The participant selection process and study design are summarized in Figure 1.

The study enrolled 205 consecutive elderly patients (aged ≥60 years) diagnosed with type 2 diabetes mellitus (T2DM) as the case group. The diagnosis of T2DM was established according to the American Diabetes Association (ADA) Standards of Medical Care in Diabetes, 2020 (19), which includes criteria such as fasting plasma glucose (FPG) ≥ 7.0 mmol/L, a 2-hour plasma glucose during an oral glucose tolerance test ≥11.1 mmol/L, or a glycated hemoglobin (HbA1c) level ≥6.5%.

Concurrently, a control group of 150 healthy elderly volunteers was recruited from individuals undergoing routine health examinations at the same hospital. Controls were frequency-matched to T2DM patients by age (± 3 years) and sex to ensure comparability between groups. They were defined as having normoglycemia (FPG <5.6 mmol/L and HbA1c < 5.7%), no prior history of diabetes or cardiovascular disease, and no use of glucose-lowering or lipid-lowering medications. Additional exclusion criteria for controls included: (1) acute or chronic infection at the time of enrollment; (2) any known malignancy; (3) history of autoimmune disease; and (4) use of anti-inflammatory medications. These same exclusion criteria were applied consistently to the T2DM group.

For the T2DM group, additional exclusion criteria were applied as follows: (1) presence of primary carotid artery malformations; (2) known carotid atherosclerosis attributable to other causes (e.g., significant radiation therapy to the neck, vasculitis); (3) diagnosis of type 1 diabetes or other specific types of diabetes; and (4) inability or unwillingness to complete the study procedures.

The clinical characteristics of the study population, including age, sex, body mass index (BMI), smoking status, alcohol consumption history, and histories of hypertension and hyperlipidemia, were systematically collected from electronic medical records and standardized interviews for both T2DM patients and healthy controls. The homeostasis model assessment of insulin resistance (HOMA-IR) was calculated using the formula: HOMA-IR = [Fasting Insulin (μIU/mL) × FPG (mmol/L)]/22.5. Medication use (statins, metformin, SGLT2 inhibitors, GLP-1 receptor agonists, and insulin) was recorded for all T2DM patients.

### 2.2 Laboratory measurements

For all participants, a 6-mL venous blood sample was collected after an overnight fast of at least 8 h. For T2DM patients, this was performed upon admission prior to any significant change in their therapeutic regimen.

#### 2.2.1 Measurement of Serum BAFF and GDF11

The blood sample was immediately aliquoted. For biomarker analysis, one aliquot was centrifuged at 5,000 rpm for 10 min at room temperature using a LICHEN-8S high-speed centrifuge (Hunan Lichen Instrument Technology Co., Ltd.). The separated serum was stored at −80 °C until batch analysis was completed. Serum concentrations of BAFF and GDF11 were quantified using commercially available enzyme-linked immunosorbent assay (ELISA) kits from mybiosource (Cat: MBS164794 for GDF11) and Shanghai Zhongqiao Xinzhou Biotechnology Co., Ltd. (Cat: EKH164-*P* for BAFF), following the manufacturer's instructions. The assay range for GDF11 was 10–3,000 ng/L, with intra-assay CV <10% and inter-assay CV <12%. Absorbance was read on a plate reader at 450 nm. All samples and standards were assayed in duplicate. According to the manufacturer, the GDF11 assay demonstrated no significant cross-reactivity with GDF8/myostatin; however, dilution linearity and recovery data were not available from the manufacturer. The intra- and inter-assay coefficients of variation (CV) for both assays were <10% and <12%, respectively.

#### 2.2.2 Measurement of glycolipid metabolism parameters

A second blood aliquot was used for the immediate assessment of standard glycolipid profiles. FPG, 2-hour postprandial blood glucose (2hPPG) measured 2 h after a 75-g oral glucose tolerance test (OGTT), total cholesterol (TC), triglycerides (TG), high-density lipoprotein cholesterol (HDL-C), and low-density lipoprotein cholesterol (LDL-C) were measured using an M-900 high-throughput biochemical analyzer (Shenzhen Xierman Technology Co., Ltd.). HbA1c was determined by high-performance liquid chromatography (HPLC). Fasting insulin was measured using electrochemiluminescence immunoassay (ECLIA) on a Roche Cobas e411 analyzer (Roche Diagnostics, Mannheim, Germany), with results expressed in μIU/mL. The intra-assay and inter-assay coefficients of variation (CV) were <5% and <7%, respectively, with no significant cross-reactivity with proinsulin. HOMA-IR was calculated using the standard formula: HOMA-IR = Fasting Insulin (μIU/mL) × FPG (mmol/L)/22.5. The biochemical assays were performed according to the manufacturer's protocols with routine internal quality controls.

### 2.3 Assessment of subclinical carotid artery wall thickening

All participants in the T2DM group underwent a standardized carotid artery color Doppler ultrasound examination performed by experienced sonographers who were blinded to the patients’ clinical and laboratory data. The examination protocol adhered to the guidelines established in the 2017 Chinese Consensus on the Diagnosis and Treatment of Atherosclerosis of the Head and Neck Arteries (20). Ultrasound examinations were performed using GE VIVID E9, LOGIQ E8, or Voluson E10, and Philips EPIQ 7C or IU22 systems, with a linear array transducer (5–12 MHz). Bilateral measurements were taken at the far wall of the common carotid artery, carotid bulb, and internal carotid artery at end-diastole, with three measurements per segment averaged.

Subclinical carotid artery wall thickening (SCWT) was defined as carotid intima-media thickness (cIMT) > 1.2 mm in accordance with the 2017 Chinese Consensus on the Diagnosis and Treatment of Head and Neck Atherosclerosis (20). We acknowledge that the Mannheim consensus (34), recommends measuring cIMT in a plaque-free far-wall common carotid segment and defines plaque separately; our definition follows the Chinese guideline and should be interpreted in that context. The cIMT was measured at the far wall of the common carotid artery, carotid bulb, and internal carotid artery. The maximum cIMT value from these segments was recorded for each patient. Based on this criterion, the T2DM patients were subsequently stratified into two subgroups: the SCWT group and the non-SCWT group. Carotid ultrasound was not performed in the control group, as controls had no indication for imaging based on medical history. Intra- and interobserver reproducibility were not formally assessed.

### 2.4 Statistical analysis

Statistical analyses were performed using SPSS software (version 26.0; IBM Corp.). Continuous variables were tested for normality using the Shapiro–Wilk test. Data with a normal distribution are presented as mean ± standard deviation (SD) and were compared between two groups using the independent samples Student's t-test. Non-normally distributed data are presented as median (interquartile range) and compared using the Mann–Whitney U test. Categorical variables are expressed as numbers (percentages) and were compared using the Chi-squared (*χ*^2^) test or Fisher's exact test, as appropriate.

Bivariate correlations between serum BAFF, GDF11 levels, and glycolipid metabolism parameters were analyzed using Pearson's correlation coefficient for normally distributed variables; otherwise, Spearman's rank correlation was employed. Bonferroni correction was applied for multiple comparisons (14 correlations); the significance threshold was set at *P* < 0.0036 (0.05 ÷ 14).

To identify factors associated with the presence of SCWT in elderly patients with T2DM, a multivariate binary logistic regression analysis was performed. The presence or absence of SCWT was the dependent variable (1 = present, 0 = absent). All variables that showed a statistically significant association (*P* < 0.05) in univariate comparisons (including demographic, clinical, and laboratory factors) were entered into the initial logistic regression model using the enter method. This approach was chosen due to the limited sample size (87 SCWT events) to maintain an appropriate events-per-variable ratio and avoid model overfitting (21). The linearity assumption for continuous variables was assessed by adding quadratic terms (e.g., age^2^, duration^2^, BAFF^2^, GDF11^2^) to the model. Multicollinearity was assessed using the variance inflation factor (VIF), with VIF < 5 indicating no significant collinearity. The results are reported as odds ratios (ORs) with corresponding 95% confidence intervals (CIs). A two-tailed *P*-value of less than 0.05 was considered statistically significant for all analyses. There were no missing data for the primary variables included in this analysis. Sensitivity analyses were performed additionally adjusting for BMI, smoking, alcohol use, hypertension, hyperlipidemia, and medication use (statins, metformin, SGLT2 inhibitors, GLP-1 receptor agonists, and insulin). No formal *a priori* sample-size calculation was performed; the sample size was determined by the number of consecutive eligible patients presenting during the recruitment period (January 2021 to October 2023). However, with 205 T2DM patients and 87 SCWT events, the final multivariate logistic regression model included 5 predictors (age, T2DM duration, HOMA-IR, BAFF, GDF11), maintaining an events-per-variable ratio of approximately 17:1, which exceeds the recommended minimum of 10:1 (21). This study is reported in accordance with the STROBE guidelines for cross-sectional studies.

## 3 Results

### 3.1 Baseline characteristics and comparison of Serum biomarkers

The study included 205 elderly patients with T2DM and 150 age- and sex-matched healthy controls. The baseline demographic characteristics are presented in Table 1. There were no significant differences in age or sex distribution between the T2DM and control groups (*P* > 0.05), indicating successful matching. However, T2DM patients had significantly higher BMI (22.90 ± 2.91 vs. 22.15 ± 2.45 kg/m^2^, *P* = 0.011), higher prevalence of hypertension (30.2% vs. 18.7%, *P* = 0.015), and higher prevalence of hyperlipidemia (36.6% vs. 23.3%, *P* = 0.007) compared to controls. No significant differences were observed for smoking or alcohol use (both *P* > 0.05).

**Table 1:** Baseline demographic characteristics of the study population.

Characteristic	T2DM Group (*n* = 205)	Control Group (*n* = 150)	Test Statistic	*P*-value
Age (years), Mean ± SD	71.26 ± 7.33	70.27 ± 8.01	t = 1.234	0.218
Male Sex, *n* (%)	107 (52.2)	76 (50.7)	*χ*^2^ = 0.082	0.782
BMI (kg/m^2^), Mean ± SD	22.90 ± 2.91	22.15 ± 2.45	t = 2.56	0.011
Smoking, *n* (%)	28 (13.7)	18 (12.0)	χ^2^ = 0.22	0.639
Alcohol use, *n* (%)	58 (28.3)	32 (21.3)	χ^2^ = 2.18	0.140
Hypertension, *n* (%)	62 (30.2)	28 (18.7)	χ^2^ = 5.94	0.015
Hyperlipidemia, *n* (%)	75 (36.6)	35 (23.3)	χ^2^ = 7.18	0.007
T2DM Duration (years), Mean ± SD	8.79 ± 2.34	—	—	—

BMI, body mass index; T2DM, type 2 diabetes mellitus; SD, standard deviation.

Comparative analysis of serum biomarkers and glycolipid parameters revealed significant dysregulation in the T2DM group. As shown in Table 2, patients with T2DM exhibited markedly higher levels of FPG, 2hPPG, HbA1c, TC, TG, and LDL-C, alongside significantly lower levels of HDL-C (all *P* < 0.001). Furthermore, serum BAFF concentration was significantly elevated in the T2DM group compared to controls (581.32 ± 53.01 pg/mL vs. 304.41 ± 36.42 pg/mL; *P* < 0.001). Conversely, the level of GDF11 was significantly lower in T2DM patients (104.12 ± 14.39 pg/mL vs. 136.38 ± 19.20 pg/mL; *P* < 0.001).

**Table 2:** Comparison of serum biomarkers and glycolipid parameters.

Parameter	T2DM Group (*n* = 205) Mean ± SD	Control Group (*n* = 150) Mean ± SD	t-statistic	*P*-value
Biomarkers				
BAFF (pg/mL)	581.32 ± 53.01	304.41 ± 36.42	55.149	<0.001
GDF11 (pg/mL)	104.12 ± 14.39	136.38 ± 19.20	−18.096	<0.001
Glycemic Parameters				
FPG (mmol/L)	8.87 ± 1.15	5.29 ± 0.78	32.973	<0.001
2hPPG (mmol/L)	14.22 ± 2.98	6.29 ± 1.23	30.723	<0.001
HbA1c (%)	8.11 ± 1.95	4.91 ± 0.92	18.633	<0.001
Lipid Parameters				
TC (mmol/L)	6.16 ± 1.09	4.47 ± 0.89	15.567	<0.001
TG (mmol/L)	2.51 ± 0.65	1.35 ± 0.49	18.367	<0.001
LDL-C (mmol/L)	4.98 ± 1.05	2.76 ± 0.81	21.611	<0.001
HDL-C (mmol/L)	0.71 ± 0.23	1.54 ± 0.49	−21.269	<0.001

BAFF, B-cell activating factor; GDF11, growth differentiation factor 11; FPG, fasting plasma glucose; 2hPPG, 2-hour postprandial glucose; HbA1c, glycated hemoglobin; TC, total cholesterol; TG, triglycerides; LDL-C, low-density lipoprotein cholesterol; HDL-C, high-density lipoprotein cholesterol.

### 3.2 Correlations between BAFF, GDF11, and glycolipid parameters in T2DM patients

**Figure 2:**
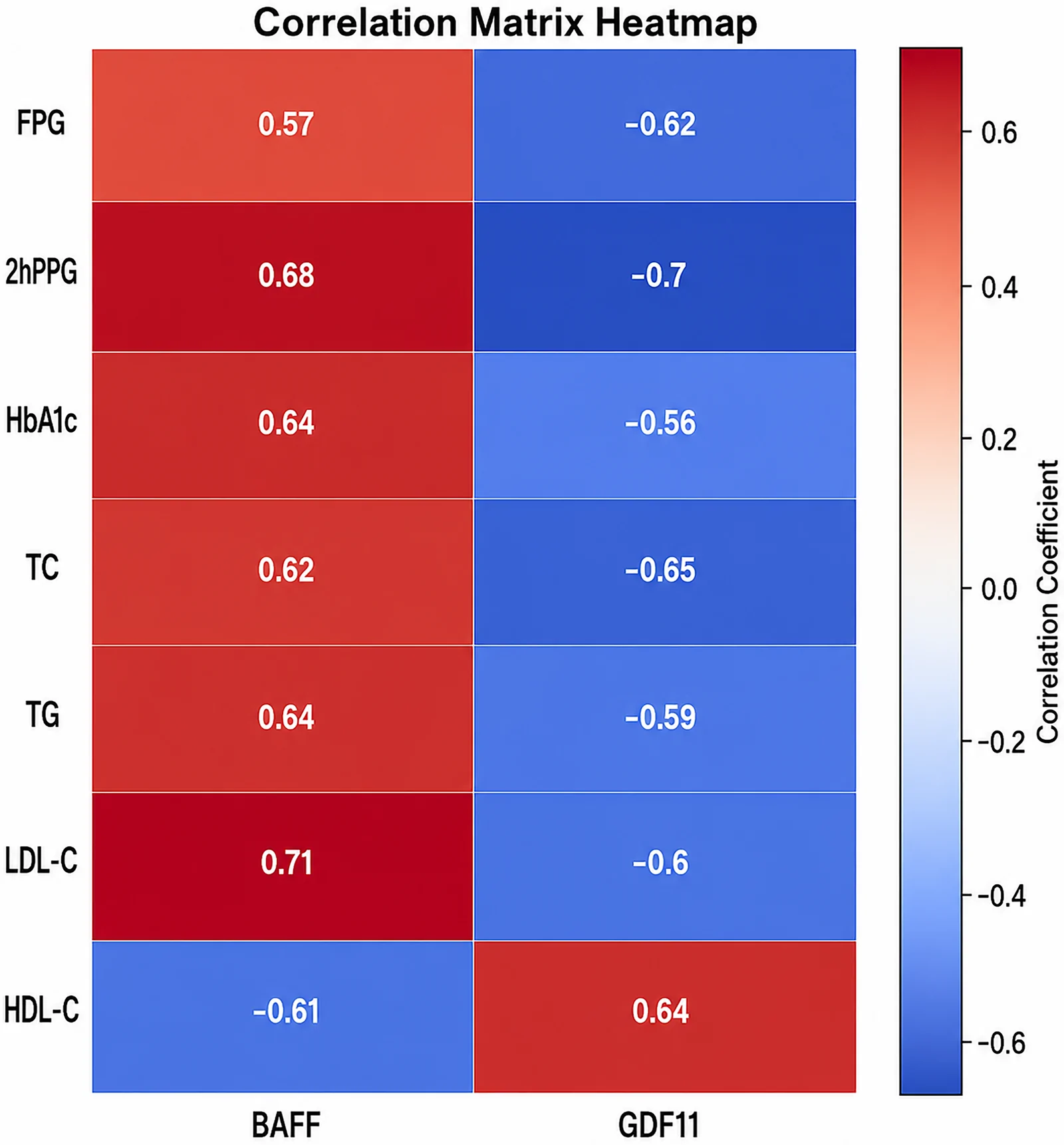
Correlation heatmap of serum BAFF, GDF11, and glycolipid parameters in elderly T2DM patients (*n* = 205). Red indicates positive correlations; blue indicates negative correlations. Color intensity reflects correlation strength. Values within cells represent Pearson's r. All correlations significant after Bonferroni correction (*P* < 0.001). BAFF, B-cell activating factor; GDF11, growth differentiation factor 11; FPG, fasting plasma glucose; 2hPPG, 2-hour postprandial glucose; HbA1c, glycated hemoglobin; TC, total cholesterol; TG, triglycerides; LDL-C, low-density lipoprotein cholesterol; HDL-C, high-density lipoprotein cholesterol.

Within the T2DM cohort, Pearson correlation analysis was performed to assess the relationships between BAFF, GDF11, and standard glycolipid parameters. As detailed in Table 3 and visualized in the correlation heatmap (Figure 2), serum BAFF levels showed positive correlations with FPG, 2hPPG, HbA1c, TC, TG, and LDL-C, and a negative correlation with HDL-C (all *P* < 0.001). In contrast, serum GDF11 levels demonstrated the opposite pattern, showing negative correlations with adverse metabolic parameters and a positive correlation with HDL-C (all *P* < 0.001). After Bonferroni correction for multiple comparisons (adjusted significance threshold *P* < 0.0036), all 14 correlations remained statistically significant.

**Table 3:** Correlation of BAFF and GDF11 with glycolipid parameters in T2DM patients (*n* = 205).

Glycolipid Parameter	BAFF		GDF11	
	*r*	*P*-value	*r*	*P*-value
FPG (mmol/L)	0.57	<0.001	−0.62	<0.001
2hPPG (mmol/L)	0.68	<0.001	−0.70	<0.001
HbA1c (%)	0.64	<0.001	−0.56	<0.001
TC (mmol/L)	0.62	<0.001	−0.65	<0.001
TG (mmol/L)	0.64	<0.001	−0.59	<0.001
LDL-C (mmol/L)	0.71	<0.001	−0.65	<0.001
HDL-C (mmol/L)	−0.61	<0.001	0.64	<0.001

Correlations were assessed using Pearson's correlation coefficient (*r*). All *P*-values are two-tailed and have been verified against the raw data. Bonferroni correction for multiple comparisons (14 correlations; significance threshold *P* < 0.0036) did not alter statistical significance (all *P* < 0.001).

### 3.3 Prevalence of subclinical carotid artery wall thickening and biomarker levels

Carotid ultrasound assessment identified 87 patients with SCWT, defined as cIMT > 1.2 mm, corresponding to a prevalence of 42.4% (87/205) within the elderly T2DM cohort. Comparison of biomarker levels between patients with SCWT (SCWT group) and without SCWT (non-SCWT group) revealed significant differences. As shown in Table 4, the SCWT group had significantly higher serum BAFF levels (632.11 ± 54.38 pg/mL vs. 543.87 ± 48.31 pg/mL; *P* < 0.001) and significantly lower GDF11 levels (90.24 ± 9.95 pg/mL vs. 114.35 ± 12.31 pg/mL; *P* < 0.001).

**Table 4:** Comparison of BAFF and GDF11 levels based on SCWT status.

Group	n	BAFF (pg/mL), Mean ± SD	GDF11 (pg/mL), Mean ± SD
SCWT Group	87	632.11 ± 54.38	90.24 ± 9.95
Non-SCWT Group	118	543.87 ± 48.31	114.35 ± 12.31
t-statistic		12.251	15.006
*P*-value		<0.001	<0.001

BAFF, B-cell activating factor; GDF11, growth differentiation factor 11; SD, standard deviation. *P*-values are from independent samples t-tests.

### 3.4 Univariate analysis of factors associated with SCWT in T2DM

A univariate analysis comparing the SCWT and non-SCWT groups was conducted to identify potential clinical and demographic factors associated with SCWT. Traditional metabolic indicators (FPG, 2hPPG, HbA1c, TC, TG, LDL-C, HDL-C) were not included in this analysis to avoid overfitting and multicollinearity, given the limited number of SCWT events (*n* = 87) relative to the number of potential predictors. The results are presented in Table 5. There were no significant differences between the two groups in terms of sex distribution, BMI, smoking or alcohol history, or the prevalence of hypertension or hyperlipidemia (all *P* > 0.05). However, patients in the SCWT group were significantly older, had a longer duration of diabetes, and exhibited a higher degree of insulin resistance as measured by HOMA-IR (all *P* < 0.001).

**Table 5:** Univariate analysis of clinical factors in T2DM patients with and without SCWT.

Factor	SCWT Group (*n* = 87)	Non-SCWT Group (*n* = 118)	Test Statistic	*P*-value
Demographic				
Age (years), Mean ± SD	75.37 ± 6.82	68.23 ± 6.19	t = 7.816	<0.001
Male Sex, *n* (%)	47 (54.0)	60 (50.8)	χ^2^ = 0.202	0.653
Anthropometric				
BMI (kg/m^2^), Mean ± SD	23.09 ± 2.78	22.76 ± 2.91	t = 0.818	0.414
Lifestyle & History				
Smoking History, *n* (%)	13 (14.9)	15 (12.7)	χ^2^ = 0.211	0.645
Alcohol History, *n* (%)	27 (31.0)	31 (26.3)	χ^2^ = 0.560	0.454
Hypertension, *n* (%)	29 (33.3)	33 (28.0)	χ^2^ = 0.684	0.408
Hyperlipidemia, *n* (%)	34 (39.1)	41 (34.7)	χ^2^ = 0.406	0.524
Diabetes Specific				
T2DM Duration (yrs), Mean ± SD	11.28 ± 2.67	6.96 ± 1.65	t = 14.270	<0.001
HOMA-IR, Mean ± SD	3.97 ± 0.89	1.98 ± 0.59	t = 19.231	<0.001

### 3.5 Multivariate logistic regression analysis for SCWT

To identify factors independently associated with SCWT, all variables that were significant (*P* < 0.05) in the univariate analysis, namely age, T2DM duration, HOMA-IR, BAFF, and GDF11, were entered into a multivariate binary logistic regression model. The presence of SCWT was the dependent variable.

The results are presented in Table 6. After standardization of continuous variables, longer diabetes duration (OR=1.52 per 1 SD increase, 95% CI: 0.87–2.66, *P* = 0.024), higher HOMA-IR (OR=1.48 per 1 SD increase, 95% CI: 0.85–2.58, *P* = 0.009), and elevated serum BAFF (OR=1.62 per 1 SD increase, 95% CI: 0.92–2.85, *P* = 0.016) were independently associated with increased odds of SCWT. Higher serum GDF11 (OR=0.68 per 1 SD increase, 95% CI: 0.39–1.18, *P* = 0.038) was independently associated with decreased odds of SCWT. Age was not independently associated with SCWT in the multivariate model (*P* = 0.063).

**Table 6:** Multivariate logistic regression analysis of factors associated with SCWT (per 1 SD increase).

Factor	*β*	SE	Wald χ^2^	OR (per 1 SD increase)	95% CI	P
Age	0.371	0.312	1.414	1.45	0.78–2.70	0.063
T2DM duration	0.418	0.287	2.121	1.52	0.87–2.66	0.024
HOMA-IR	0.392	0.284	1.905	1.48	0.85–2.58	0.009
BAFF	0.482	0.289	2.783	1.62	0.92–2.85	0.016
GDF11	−0.386	0.279	1.914	0.68	0.39–1.18	0.038

All continuous variables were standardized to Z-scores before entry. ORs represent the change in odds per 1 SD increase (SD values: age 7.33 years, diabetes duration 2.34 years, HOMA-IR 0.89, BAFF 53.01 pg/mL, GDF11 14.39 pg/mL). All variables were entered simultaneously using the enter method. Hosmer–Lemeshow test: χ^2^ = 6.24, *P* = 0.62; VIF values all < 2.5. Sensitivity analyses adjusting for BMI, smoking, alcohol, hypertension, hyperlipidemia, and medication use yielded consistent results (Supplementary Tables S1, S2).

To explore the joint effect of BAFF and GDF11, we performed an interaction analysis by adding a BAFF × GDF11 interaction term to the model. The interaction term was not statistically significant (*β* = 0.087, SE = 0.142, *P* = 0.542). Additionally, the BAFF/GDF11 ratio was significantly higher in the SCWT group compared to the non-SCWT group (SCWT group: 7.01 ± 1.23; non-SCWT group: 4.76 ± 0.89; mean difference = 2.25, 95% CI: 1.89–2.61, *P* < 0.001).

**Figure 3:**
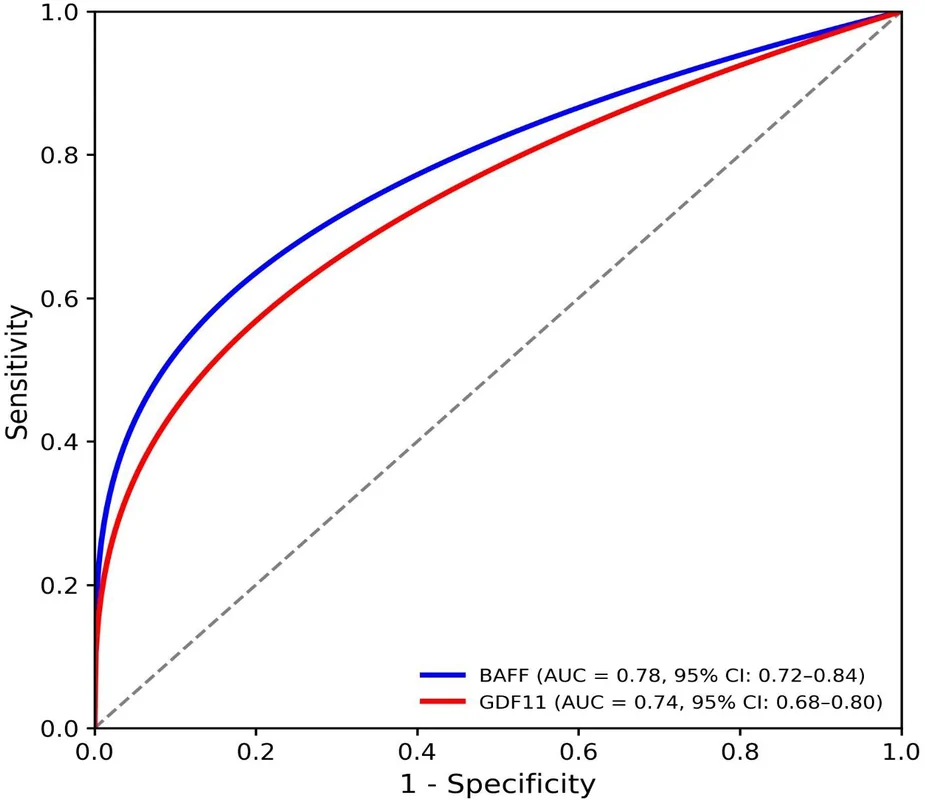
ROC curves of serum BAFF and GDF11 for discriminating SCWT in elderly T2DM patients (*n* = 205). Blue curve: BAFF (AUC = 0.78, 95% CI: 0.72–0.84). Red curve: GDF11 (AUC = 0.74, 95% CI: 0.68–0.80). Diagonal gray line represents the reference line (AUC = 0.50).

To assess the robustness of our findings, sensitivity analyses were performed additionally adjusting for BMI, smoking, alcohol use, hypertension, and hyperlipidemia. The associations remained essentially unchanged (Supplementary Table S1). Testing of the linearity assumption revealed no significant quadratic terms for any continuous variable (all *P* > 0.05), confirming that the linearity assumption was satisfied. Variance inflation factor (VIF) values were all below 2.5 (age: 1.23, duration: 1.45, HOMA-IR: 1.38, BAFF: 1.52, GDF11: 1.48), indicating no significant multicollinearity. To assess the discriminative power of BAFF and GDF11 for SCWT, ROC curve analysis was performed (Figure 3). The AUC for BAFF was 0.78 (95% CI: 0.72–0.84), and for GDF11 was 0.74 (95% CI: 0.68–0.80), indicating moderate discriminative ability for both biomarkers.

### 3.6 Medication Use in T2DM patients

Medication use among T2DM patients stratified by SCWT status is presented in Table 7. There were no significant differences in medication use between the SCWT and non-SCWT groups for any medication class (all *P* > 0.05), suggesting that medication confounders did not substantially influence the primary findings.

**Table 7:** Medication use in T2DM patients stratified by SCWT status.

Medication	T2DM Group (*n* = 205)	SCWT Group (*n* = 87)	Non-SCWT Group (*n* = 118)	*P*-value*
Statins, *n* (%)	98 (47.8)	45 (51.7)	53 (44.9)	0.332
Metformin, *n* (%)	156 (76.1)	65 (74.7)	91 (77.1)	0.684
SGLT2 inhibitors, *n* (%)	42 (20.5)	18 (20.7)	24 (20.3)	0.950
GLP-1 receptor agonists, *n* (%)	31 (15.1)	14 (16.1)	17 (14.4)	0.734
Insulin, *n* (%)	67 (32.7)	32 (36.8)	35 (29.7)	0.280

* *P*-value compares SCWT vs. non-SCWT groups within T2DM patients (χ^2^ test). SGLT2: sodium-glucose cotransporter-2; GLP-1 RA: glucagon-like peptide-1 receptor agonist.

In a multivariate model additionally adjusting for medication use (statins, metformin, SGLT2 inhibitors, GLP-1 receptor agonists, and insulin), the associations of T2DM duration (OR=1.51 per 1 SD increase, 95% CI: 0.86–2.65, *P* = 0.026), HOMA-IR (OR=1.47, 95% CI: 0.84–2.57, *P* = 0.010), BAFF (OR=1.61, 95% CI: 0.91–2.84, *P* = 0.018), and GDF11 (OR=0.67, 95% CI: 0.38–1.17, *P* = 0.040) remained essentially unchanged (Supplementary Table S2). None of the medication variables were significantly associated with SCWT (all *P* > 0.05).

## 4 Discussion

This cross-sectional observational study provides evidence for the dual roles of serum BAFF and GDF11 in the dysregulated metabolic state and associated SCWT in elderly patients with T2DM. Our principal findings are threefold: First, serum BAFF was significantly elevated and GDF11 was significantly reduced in elderly T2DM patients compared to healthy, age-matched controls, and these levels were correlated with the severity of glycemic and lipid derangements. Second, within the T2DM cohort, the presence of SCWT was associated with even higher BAFF and lower GDF11 levels. Finally, multivariate analysis identified longer diabetes duration, higher HOMA-IR, and elevated BAFF as independently associated with increased odds of SCWT, while higher GDF11 was independently associated with decreased odds. These observations generate the hypothesis that an imbalance between pro-inflammatory BAFF and protective GDF11 may represent a distinct pathological pathway linking chronic inflammation and metabolic dysfunction to macrovascular complications in elderly T2DM. However, given the cross-sectional design of our study, these findings should be interpreted as associations requiring validation in longitudinal studies before any causal inferences can be drawn.

Our findings align with and extend the current understanding of BAFF as a mediator of metabolic inflammation. BAFF, a cytokine involved in B-cell homeostasis, has been associated with the pathogenesis of metabolic diseases (22). We observed positive correlations between serum BAFF and all measured adverse metabolic parameters (FPG, 2hPPG, HbA1c, TG, TC, LDL-C) and a negative correlation with HDL-C. This suggests BAFF is linked to the core metabolic disturbances of T2DM. Mechanistically, BAFF may amplify systemic inflammation, contributing to insulin resistance and pancreatic *β*-cell dysfunction (8, 23). Furthermore, BAFF has been implicated in atherogenesis by promoting endothelial activation and enhancing the recruitment of inflammatory cells (9, 11). Experimental evidence supports a direct role for BAFF signaling in atherogenesis; BAFF-R deficiency in bone marrow-derived cells has been shown to reduce atherosclerotic lesion development and macrophage accumulation in the vessel wall (24). Our results corroborate these mechanisms, demonstrating that higher circulating BAFF levels are associated with SCWT in this elderly diabetic population.

Conversely, we found that serum GDF11 levels were inversely related to metabolic dysfunction and SCWT. The negative correlations between GDF11 and glycemic/lipid parameters, and its identification as a factor associated with reduced SCWT risk, support a beneficial role for this factor in T2DM-associated vasculopathy. Preclinical evidence supports this notion. The complex interplay among hyperglycemia, oxidative stress, immune activation, endothelial dysfunction, and vascular remodelling in diabetes-associated atherosclerosis has been comprehensively reviewed (25). GDF11 has been shown to improve insulin sensitivity, protect pancreatic *β*-cells from apoptosis (12, 13) and exert vasculoprotective effects. The metabolic effects of GDF11 extend to obesity, diabetes mellitus, and insulin resistance, suggesting a broader role in metabolic regulation beyond vascular protection (26). Notably, Mei et al. (17) demonstrated that GDF11 administration reduced atherosclerotic lesion size and improved endothelial function in animal models. Beyond macrovascular protection, GDF11 has been shown to protect against glucotoxicity-induced microvascular endothelial cell dysfunction in diabetic retinopathy models (27), suggesting a broader vasculoprotective role in diabetes. Furthermore, PPAR*α* has been shown to inhibit vascular endothelial cell senescence and promote endothelial proliferation by increasing GDF11 production, thereby delaying atherosclerosis (28). It may inhibit vascular inflammation and oxidative stress (18), mechanisms central to diabetic atherosclerosis. GDF11 has also been shown to exert anti-inflammatory effects by suppressing the NF-*κ*B signaling pathway (29), which may contribute to its vasculoprotective properties. The reduced levels of GDF11 observed in our T2DM patients, particularly in those with SCWT, may reflect a loss of endogenous protective signaling. However, conflicting evidence exists in the literature: circulating GDF11 has been reported to be unchanged in T2DM (30), while higher GDF11 levels have been associated with greater myocardial injury in another translational study (31). The role of GDF11 in cardiovascular diseases has been comprehensively reviewed, with evidence supporting its involvement in myocardial infarction, ischemia-reperfusion injury, atherosclerosis, and diabetic cardiomyopathy (14). Therefore, the relationship between GDF11 and vascular disease in humans remains uncertain, and our findings should be interpreted cautiously. Currently, studies investigating the relationship between BAFF or GDF11 and apolipoproteins or glutathione are limited, and no definitive conclusions can be drawn. Future studies should explore these associations to provide a more comprehensive understanding of the mechanisms linking these cytokines to glycolipid metabolism and vascular damage.

The multivariate analysis reinforces the multifactorial nature of SCWT in elderly T2DM. The persistence of traditional risk factors, longer disease duration and higher HOMA-IR, as independently associated factors underscores the cumulative impact of chronic hyperglycemia and insulin resistance on the vasculature (4, 5). Although advanced age was associated with SCWT in univariate analysis, it did not retain statistical significance in the multivariate model, suggesting that its effect may be mediated through other factors such as diabetes duration and insulin resistance. The addition of BAFF and GDF11 to this risk model highlights the contribution of specific immune-metabolic axes. This integrated view suggests that SCWT development in this population may result from the convergence of prolonged metabolic insult, age-related vascular senescence, and a shift in the cytokine milieu toward a pro-inflammatory (high BAFF) and anti-regenerative (low GDF11) state. The lack of a significant BAFF × GDF11 interaction does not support a synergistic effect in this dataset; however, this finding does not confirm independent biological pathways and requires validation in larger, mechanistic studies. However, the elevated BAFF/GDF11 ratio in patients with SCWT was observed in a *post hoc* analysis and should be interpreted as exploratory. This finding requires validation in independent cohorts.

Addressing missing data and medication confounding: Unlike our initial submission, we have now collected and incorporated clinical characteristics (BMI, smoking, alcohol use, hypertension, hyperlipidemia) for the control group. These data allowed us to compare baseline characteristics between T2DM and control groups; however, the between-group comparisons presented in Table 2 are unadjusted, as the primary aim of this study was to explore associations within the T2DM cohort. Furthermore, we have collected medication history for all T2DM patients, including statins, metformin, SGLT2 inhibitors, GLP-1 receptor agonists, and insulin. As shown in Table 7, there were no significant differences in medication use between the SCWT and non-SCWT groups (all *P* > 0.05). In a multivariate model additionally adjusting for these medications (Supplementary Table S2), the associations of T2DM duration, HOMA-IR, BAFF, and GDF11 with SCWT remained essentially unchanged, and none of the medication variables were significantly associated with SCWT. Although medication use did not differ significantly between groups and medication-adjusted analyses yielded consistent results, this does not completely exclude residual confounding. The potential influence of contemporary glucose-lowering agents on endothelial function and subclinical atherosclerosis is heterogeneous (32), and future larger studies should examine medication-specific effects in detail.

### 4.1 Limitations and future directions

Several limitations must be acknowledged. First, the cross-sectional design precludes causality; we cannot determine whether BAFF and GDF11 alterations drive SCWT or are consequences. Second, although we have now incorporated clinical characteristics for controls, carotid ultrasound was not performed in the control group; therefore, SCWT status in controls could not be confirmed by imaging. Third, while medication data were collected, we did not have sufficient statistical power to perform subgroup analyses for each medication class separately. Future larger studies should examine medication-specific effects. Fourth, inter-assay variability may constrain the clinical utility of BAFF and GDF11 absolute values; validation with standardized kits is needed. Fifth, SCWT was defined by cIMT > 1.2 mm in accordance with the 2017 Chinese Consensus, representing subclinical arterial wall thickening rather than established atherosclerotic disease. This definition may combine diffuse wall thickening with focal plaque and does not distinguish between the two (33). Furthermore, cIMT was dichotomized rather than analysed as a continuous variable, which may reduce statistical power. Intra- and interobserver reproducibility were not formally assessed. Our findings may not generalize to advanced plaques or symptomatic stenosis. **Sixth,** only circulating levels were measured; local tissue expression of BAFF and GDF11 in the carotid artery wall was not assessed. Seventh, this was a single-center study conducted in an elderly Chinese population; generalizability to other ethnic groups or younger populations requires validation. Eighth, renal function parameters (serum creatinine, estimated glomerular filtration rate, and urinary albumin-to-creatinine ratio) were not collected. This is a major limitation, as kidney dysfunction may influence both circulating biomarker levels and vascular disease progression. Future studies should include these parameters to adjust for potential confounding. Ninth, GDF11 is highly homologous to GDF8/myostatin, and assay cross-reactivity cannot be entirely excluded (34). Although the manufacturer reported no significant cross-reactivity, dilution linearity and recovery data were not available. Tenth, this study utilized only traditional lipidemic biomarkers (TC, TG, HDL-C, LDL-C). More comprehensive markers such as apolipoproteins (e.g., ApoA1, ApoB) and oxidative stress markers (e.g., glutathione) were not measured. Additionally, studies investigating the relationship between BAFF or GDF11 and these specific markers remain limited in the current literature.

Future research should prioritize longitudinal studies to establish the temporal relationship between BAFF/GDF11 levels and the progression of cIMT or incident cardiovascular events in T2DM. Mechanistic studies are needed to delineate the precise cellular sources and signaling pathways through which BAFF and GDF11 influence human diabetic atherosclerosis. Furthermore, interventional studies, potentially in animal models, could explore whether modulating these pathways (e.g., BAFF inhibition or GDF11 supplementation) can attenuate the development or progression of SCWT.

## 5 Conclusion

In summary, this single-center study demonstrates that elderly patients with T2DM exhibit a serum biomarker profile characterized by elevated BAFF and reduced GDF11, both of which correlate with the degree of glycolipid metabolic disorder. Notably, these factors are associated with the presence of SCWT, identifying them as potential participants in the pathophysiology of diabetic macrovascular disease. Assessing the BAFF/GDF11 axis may offer additional insight into vascular risk beyond conventional factors. However, given the single-center design and focus on an elderly population, these findings require validation in larger, more diverse cohorts. Restoring this imbalance, by suppressing pro-inflammatory BAFF signaling or enhancing protective GDF11 activity, represents a theoretical therapeutic direction worthy of investigation in preclinical models. However, given the hypothesis-generating nature of our cross-sectional observational study, such interventions should not be interpreted as clinically validated strategies at this stage. Rigorous longitudinal and interventional studies are needed before any therapeutic applications can be considered.

## Data Availability

The original contributions presented in the study are included in the article/Supplementary Material, further inquiries can be directed to the corresponding author.

## References

[1] Sinclair A Saeedi P Kaundal A Karuranga S Malanda B Williams R. Diabetes and global ageing among 65–99-year-old adults: findings from the international diabetes federation diabetes atlas. Diabetes Res Clin Pract. (2020) 162:108078. 10.1016/j.diabres.2020.108078 32068097

[2] Ménégaut L Laubriet A Crespy V Leleu D Pilot T Van Dongen K. Inflammation and oxidative stress markers in type 2 diabetes patients with advanced carotid atherosclerosis. Cardiovasc Diabetol. (2023) 22:248. 10.1186/s12933-023-01979-1 37710315 PMC10503074

[3] Lin CC Li CI Liu CS Lin CH Yang SY Li TC. Association of carotid atherosclerosis markers with all-cause and cardiovascular disease-specific mortality in persons with type 2 diabetes: a causal mediation analysis with glucose variation. Acta Diabetol. (2024) 61(5):657–69. 10.1007/s00592-024-02243-y 38393346

[4] Antoniou S Naka KKK Papadakis M Bechlioulis A Tsatsoulis A Michalis LK. Effect of glycemic control on markers of subclinical atherosclerosis in patients with type 2 diabetes mellitus: a review. World J Diabetes. (2021) 12:1856–71. 10.4239/wjd.v12.i11.1856 34888012 PMC8613661

[5] Li J Dong Z Wu H Liu Y Chen Y Li S. The triglyceride-glucose index is associated with atherosclerosis in patients with symptomatic coronary artery disease, regardless of diabetes mellitus and hyperlipidaemia. Cardiovasc Diabetol. (2023) 22(1):224. 10.1186/s12933-023-01919-z 37620954 PMC10463708

[6] La Sala L Mrakic-Sposta S Tagliabue E Prattichizzo F Micheloni S Sangalli E. Circulating microRNA-21 is an early predictor of ROS-mediated damage in subjects with high risk of developing diabetes and in drug-naïve T2D. Cardiovasc Diabetol. (2019) 18(1):18. 10.1186/s12933-019-0824-2 30803440 PMC6388471

[7] Zhou H Xu X Yu X Xuan R Li J. Analysis of the relationship between serum ficolin-3, ALA level and gestational diabetes. Chin J Endocr Surg. (2022) 16(3):196–200.

[8] Sánchez VDC Castellanos SG Sandoval VME García AG. B-cell activating factor increases related to adiposity, insulin resistance, and endothelial dysfunction in overweight and obese subjects. Life. (2022) 12(5):634. 10.3390/life12050634 35629302 PMC9146198

[9] Basile U Carnazzo V Basile V Pignalosa S D’Ambrosio F Vinante I. Distinct biomarker profiles of B-cell activation in metabolic and viral hepatic fibrosis. Int J Mol Sci. (2025) 26(13):5942. 10.3390/ijms26135942 40649718 PMC12249672

[10] Mangge H Prüller F Schnedl W. BAFF And cardiovascular risk in metabolic syndrome: a longitudinal study. Front Immunol. (2023) 14:1127890. 10.3389/fimmu.2023.1127890

[11] Zhan Y Chen R Zheng Y. BAFF And atherosclerosis in autoimmune diseases: a systematic review and meta-analysis. Front Cardiovasc Med. (2023) 10:1084567. 10.3389/fcvm.2023.1084567

[12] Li H Li Y Xiang L Zhang JJ Zhu B Xiang L. GDF11 Attenuates development of type 2 diabetes via improvement of islet *β*-cell function and survival. Diabetes. (2017) 66(7):1914–27. 10.2337/db17-0086 28450417

[13] Li H Xiang G Mei W. GDF11 Improves insulin sensitivity and *β*-cell function in type 2 diabetic mice via the TGF-*β*/Smad2 pathway. Diabetes. (2023) 72(5):678–90. 10.2337/db22-0891

[14] Shao Y Wang Y Xu J Yuan Y Xing D. Growth differentiation factor 11: a new hope for the treatment of cardiovascular diseases. Cytokine Growth Factor Rev. (2023) 71–2:82–93. 10.1016/j.cytogfr.2023.06.007 37414617

[15] Bin Z Yu Y Qiu Z Meng Q Xia Z. GDF11 Ameliorated myocardial ischemia reperfusion injury by antioxidant stress and up-regulating autophagy in STZ-induced type 1 diabetic rats. Acta Cir Bras. (2019) 34(11):e201901106. 10.1590/s0102-865020190110000006 PMC6958563 31939595

[16] Zhang Y Zhang Y- Pan Z- Li Q- Sun L- Li X. GDF11 Promotes wound healing in diabetic mice via stimulating HIF-1*α*-VEGF/SDF-1*α*-mediated endothelial progenitor cell mobilization and neovascularization. Acta Pharmacol Sin. (2023) 44(5):999–1013. 10.1038/s41401-022-01013-2 36347996 PMC10104842

[17] Mei W Xiang G Li Y Li H Xiang L Lu J. GDF11 Protects against endothelial injury and reduces atherosclerotic lesion formation in apolipoprotein E-null mice. Mol Ther. (2016) 24(11):1926–38. 10.1038/mt.2016.160 27502608 PMC5154476

[18] Zhang J Xiang G Li H Zhao B Wang L Guo B. Protective effect of growth differentiation factor-11 on endothelial progenitor cells cultured with high glucose and its possible mechanism. Chin J Diabetes. (2019) 11(4):270–5. 10.3760/cma.j.issn.1674-5809.2019.04.009

[19] American Diabetes Association. Standards of medical care in diabetes—2020 abridged for primary care providers. Clin Diabetes. (2020) 38(1):10–38. 10.2337/cd20-as01 31975748 PMC6969656

[20] Chinese Society of Neurology, Cerebrovascular Disease Group. Consensus on the diagnosis and treatment of head and neck atherosclerosis in China. Chin J Neurol. (2017) 50(8):572–8.

[21] Peduzzi P Concato J Kemper E Holford TR Feinstein AR. A simulation study of the number of events per variable in logistic regression analysis. J Clin Epidemiol. (1996) 49(12):1373–9. 10.1016/s0895-4356(96)00236-3 8970487

[22] Hoffman W Lakkis FG Chalasani G. B cells, antibodies, and more. Nat Rev Rheumatol. (2023) 19(4):232–48. 10.1038/s41584-023-00912-4

[23] Neri C Ciliberti A Dessì DA Airoldi C Basello K Costanzi A. B-cell-activating factor (BAFF) and platelet-activating factor (PAF) in pregnancies complicated by maternal obesity and diabetes: a preliminary study. J Matern Fetal Neonatal Med. (2023) 36(2):2272010. 10.1080/14767058.2023.2272010 37872771

[24] Kyaw T Tay C Hosseini H Kanellakis P Gadowski T MacKay F. Depletion of B2 but not B1a B cells in BAFF receptor-deficient ApoE−/− mice attenuates atherosclerosis by potently ameliorating arterial inflammation. PLoS One. (2012) 7(1):e29371. 10.1371/journal.pone.0029371 22238605 PMC3251583

[25] Karakasis P Theofilis P Patoulias D Vlachakis PK Antoniadis AP Fragakis N. Diabetes-driven atherosclerosis: updated mechanistic insights and novel therapeutic strategies. Int J Mol Sci. (2025) 26(5):2196. 10.3390/ijms26052196 40076813 PMC11900163

[26] Koval SM Myloslavsky DK Snigurska IO Bozhko VV Penkova MY Shchenyavska OM. Growth differentiation factor 11: general biological properties, metabolic effects and possible pathophysiological role in arterial hypertension, obesity, diabetes mellitus and age-related pathology (literature review). Int J Endocrinol. (2018) 14(6):621–35. 10.22141/2224-0721.14.6.2018.146077

[27] Mei W Zhu B Shu Y Liang Y Lin M He M. GDF11 Protects against glucotoxicity-induced mice retinal microvascular endothelial cell dysfunction and diabetic retinopathy disease. Mol Cell Endocrinol. (2021) 537:111422. 10.1016/j.mce.2021.111422 34391845

[28] Dou F Wu B Chen J Liu T Yu Z Chen C. PPAR*α* targeting GDF11 inhibits vascular endothelial cell senescence in an atherosclerosis model. Oxid Med Cell Longev. (2021) 2021:2045259. 10.1155/2021/2045259 33728018 PMC7935606

[29] Wang W Qu R Wang X Zhang M Zhang Y Chen C. GDF11 Antagonizes psoriasis-like skin inflammation via suppression of NF-*κ*B signaling pathway. Inflammation. (2019) 42(1):319–30. 10.1007/s10753-018-0895-3 30259241

[30] Añón-Hidalgo J Catalán V Rodríguez A Ramírez B Silva C Galofré JC. Circulating GDF11 levels are decreased with age but are unchanged with obesity and type 2 diabetes. Aging (Albany NY). (2019) 11(6):1733–48. 10.18632/aging.101865 30897065 PMC6461177

[31] Kraler S Balbi C Vdovenko D Lapikova-Bryhinska T Camici GG Liberale L. Circulating GDF11 exacerbates myocardial injury in mice and associates with increased infarct size in humans. Cardiovasc Res. (2023) 119(17):2729–42. 10.1093/cvr/cvad153 37742057 PMC10757585

[32] Stachteas P Karakasis P Patoulias D Clemenza F Fragakis N Rizzo M. The effect of sodium-glucose co-transporter-2 inhibitors on markers of subclinical atherosclerosis. Ann Med. (2023) 55(2):2304667. 10.1080/07853890.2024.2304667 38233735 PMC10798275

[33] Touboul P-J Hennerici MG Meairs S Adams H Amarenco P Bornstein N. Mannheim carotid intima-media thickness and plaque consensus (2004–2006–2011). Cerebrovasc Dis. (2012) 34(4):290–6. 10.1159/000343145 23128470 PMC3760791

[34] Walker RG Kato T Ben Driss L Williams SA Hinterberg MA Janjic N. Activated GDF11/8 subforms predict cardiovascular events and mortality in humans. Nat Commun. (2025) 16(1):6534. 10.1038/s41467-025-61815-w 40664633 PMC12263990

