# Supplementary material for: Serum BAFF and GDF11: cross-sectional associations with glycolipid metabolism and subclinical carotid artery wall thickening in elderly type 2 diabetes patients
Source: Front Cardiovasc Med. 2026 Aug 18;13:1891416. doi: 10.3389/fcvm.2026.1891416 (PMC13530899; doi:10.3389/fcvm.2026.1891416)
Supplement: Supplementary file 1 [file Table1.docx]

**Table S1.** Sensitivity analysis of factors associated with SCWT (per 1 SD increase).

| **Model** | **Variable** | **OR (per 1 SD increase)** | **95% CI** | **P-value** |
| --- | --- | --- | --- | --- |
| Primary Model (adjusted for age, duration, HOMA-IR) | T2DM duration | 1.52 | 0.87–2.66 | 0.024 |
|  | HOMA-IR | 1.48 | 0.85–2.58 | 0.009 |
|  | BAFF | 1.62 | 0.92–2.85 | 0.016 |
|  | GDF11 | 0.68 | 0.39–1.18 | 0.038 |
| Sensitivity Model (additional adjustment for BMI, smoking, alcohol, hypertension, hyperlipidemia) | T2DM duration | 1.53 | 0.88–2.67 | 0.025 |
|  | HOMA-IR | 1.47 | 0.84–2.57 | 0.009 |
|  | BAFF | 1.63 | 0.93–2.86 | 0.016 |
|  | GDF11 | 0.67 | 0.38–1.17 | 0.039 |
| Within-T2DM Sensitivity Model (adjusting for BMI, smoking, alcohol, hypertension, hyperlipidemia within T2DM group only) | BAFF | 1.61 | 0.91–2.84 | 0.016 |
|  | GDF11 | 0.68 | 0.39–1.18 | 0.039 |

All continuous variables were standardized to Z-scores before entry. ORs represent the change in odds per 1 standard deviation (SD) increase. SD values: age (7.33 years), diabetes duration (2.34 years), HOMA-IR (0.89), BAFF (53.01 pg/mL), GDF11 (14.39 pg/mL). Primary model adjusted for age, T2DM duration, HOMA-IR, BAFF, and GDF11. Age was not statistically significant (OR = 1.45, 95% CI: 0.78–2.70, P = 0.063) and is not shown for brevity (see Table 6). Sensitivity model additionally adjusted for BMI, smoking, alcohol use, hypertension, and hyperlipidemia despite their non-significance in univariate analysis. Within-T2DM sensitivity model was performed within the T2DM group only to assess the impact of missing control group data. All variables were entered simultaneously using the enter method.

**Table S2.** Multivariate logistic regression analysis of factors associated with SCWT adjusting for medication use (per 1 SD increase).

| **Factor** | **OR (per 1 SD increase)** | **95% CI** | **P-value** |
| --- | --- | --- | --- |
| T2DM duration | 1.51 | 0.86–2.65 | 0.026 |
| HOMA-IR | 1.47 | 0.84–2.57 | 0.010 |
| BAFF | 1.61 | 0.91–2.84 | 0.018 |
| GDF11 | 0.67 | 0.38–1.17 | 0.040 |
| Statins | 1.12 | 0.65–1.93 | 0.682 |
| Metformin | 0.95 | 0.52–1.74 | 0.872 |
| SGLT2 inhibitors | 0.98 | 0.48–2.00 | 0.956 |
| GLP-1 receptor agonists | 1.08 | 0.49–2.38 | 0.848 |
| Insulin | 1.15 | 0.62–2.13 | 0.654 |

All continuous variables were standardized to Z-scores before entry. ORs represent the change in odds per 1 standard deviation (SD) increase. SD values: T2DM duration (2.34 years), HOMA-IR (0.89), BAFF (53.01 pg/mL), GDF11 (14.39 pg/mL). Medication variables were entered as categorical variables (1 = currently taking, 0 = not taking). All variables were entered simultaneously using the enter method.
